# Enhanced Potassium-Ion Storage of the 3D Carbon Superstructure by Manipulating the Nitrogen-Doped Species and Morphology

**DOI:** 10.1007/s40820-020-00525-y

**Published:** 2020-10-27

**Authors:** Yanhua Li, Kui Xiao, Cong Huang, Jin Wang, Ming Gao, Aiping Hu, Qunli Tang, Binbin Fan, Yali Xu, Xiaohua Chen

**Affiliations:** 1 College of Materials Science and Engineering, Hunan University, Changsha, 410082 People’s Republic of China; 2 College of Materials and Chemistry Engineering, Hunan Institute of Technology, Hengyang, 421002 People’s Republic of China; 3 Zhuzhou Times New Material Technology Co., LTD, Zhuzhou, 412007 People’s Republic of China

**Keywords:** Polyimide, Nitrogen-doped, Potassium-ion battery, 3D carbon material

## Abstract

Potassium-ion batteries (PIBs) are attractive for grid-scale energy storage due to the abundant potassium resource and high energy density. The key to achieving high-performance and large-scale energy storage technology lies in seeking eco-efficient synthetic processes to the design of suitable anode materials. Herein, a spherical sponge-like carbon superstructure (NCS) assembled by 2D nanosheets is rationally and efficiently designed for K^+^ storage. The optimized NCS electrode exhibits an outstanding rate capability, high reversible specific capacity (250 mAh g^−1^ at 200 mA g^−1^ after 300 cycles), and promising cycling performance (205 mAh g^−1^ at 1000 mA g^−1^ after 2000 cycles). The superior performance can be attributed to the unique robust spherical structure and 3D electrical transfer network together with nitrogen-rich nanosheets. Moreover, the regulation of the nitrogen doping types and morphology of NCS-5 is also discussed in detail based on the experiments results and density functional theory calculations. This strategy for manipulating the structure and properties of 3D materials is expected to meet the grand challenges for advanced carbon materials as high-performance PIB anodes in practical applications.

**Electronic supplementary material:**

The online version of this article (10.1007/s40820-020-00525-y) contains supplementary material, which is available to authorised users.

## Highlights

- An eco-efficient synthetic route was developed to establish carbon superstructures with enhanced exposed nitrogen-rich active facets.
- The synergistic effect of the 3D interconnected superstructures and the high nitrogen-doping content endows the N-rich carbon superstructures (NCS-5) with not only increased potassium-ion storage capabilities but also superior rate and cycling performance.
- The regulation of the nitrogen doping types and morphology of NCS-5 is also discussed in detail based on the experiment results and density functional theory calculations.

**Electronic supplementary material:**

The online version of this article (10.1007/s40820-020-00525-y) contains supplementary material, which is available to authorised users.

## Graphical abstract

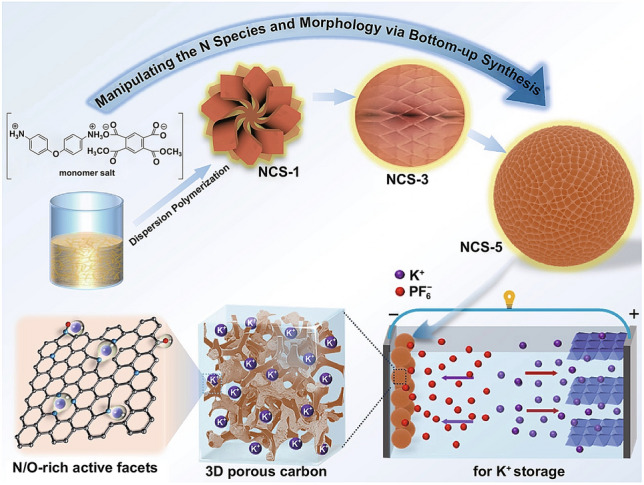

## Introduction

Developing low-cost, stable, and nontoxic rechargeable post-lithium (Li)-ion batteries, such as sodium (Na)-ion batteries, aluminum-ion batteries, and potassium-ion batteries (PIBs), is of great significance to meet the demands of large-scale energy storage systems [1–6]. In particular, PIBs are promising because of (1) the low cost and high earth abundance of potassium (K) resources [7]; (2) the low oxidation and reduction potentials of K^+^/K (− 2.936 V vs. standard hydrogen electrode (SHE)), implying that PIBs should possess a high voltage plateau and high energy density [8–10]; and (3) the weaker Lewis acidity of K^+^ than that of Li^+^, which indicates the higher mobility of K^+^ than that of Li^+^ in the equivalent solution [11]. Based on the three features, great research effort has been devoted to seeking suitable electrode materials with the goal of achieving high-performance PIBs for large-scale energy storage [12, 13]. However, an important obstacle for the rapid development of PIBs is the absence of suitable anode materials, which typically determines the bottleneck for cell performance and large-scale application. Therefore, to realize the scaling feasibility of the application, the design of anode material incorporated outstanding performance and eco-efficient synthetic processes remains to be done [14, 15].

Owing to the abundance, nontoxicity, safety, and durability, carbon-based anode materials that can satisfy the crucial characteristics of commercial electrodes are considered promising candidates for large-scale applications [16–18]. To date, a variety of carbon materials have been reported as anode materials for PIBs, such as graphite materials [19], graphene materials [26], hard carbon materials, and carbon-based composite materials [23]. The common potassium storage mechanisms in above carbon-based materials are insertion and adsorption mechanisms. However, owing to the poor diffusion ability of K^+^ with larger size, it is hard for the absolute K-intercalation into the graphite layers to achieve a high rate capacity. It is widely believed that K-adsorption onto the nanovoids and surface defects/functional groups can promote fast ion diffusion and maintain excellent structure stability. Considering the expanded interlayer spacing and adjusted defects, hard carbon materials have been widely investigated due to the kinetically favorable features for the transportation of ion and electron. Among them, doping hard carbon with nitrogen (N) is one of the most studied strategies, which can effectively improve the electrochemical performance of carbonaceous materials, including conductivity, wettability, chemical stability, and active sites for K^+^ adsorption, thus yielding a high gravimetric capacity for K^+^ storage [20–23]. Highly N-doped carbon materials are usually synthesized using an N-rich carbon precursor, which results in four common types of N-based functional groups: pyridinic-N (N-6), pyrrolic-N (N-5), quaternary-N (N-Q), and oxidized pyridinic-N (N-X) [24]. A combination of these species is considered beneficial to raise performance. In particular, density functional theory (DFT) calculations revealed that N-6 and N-5 sites have much higher K^+^ adsorption abilities than that of N-Q sites [23]. This encouraged us to develop carbonaceous materials with high N-6 and N-5 nitrogen doping levels for enhanced potassium-ion storage capability.

In addition to N-doping strategy, the construction of stable three-dimensional (3D) carbon superstructures (CSSs) constructed from the assembly of nanosheets is also an important approach to enhance the electrochemical performance of carbon materials. Although sheet-like nanomaterials with defects/functional groups are beneficial to high gravimetric capacity for K^+^ storage, some major challenges still exist for the direct use of them, such as their serious self-agglomerating tendency during electrode fabrication and low conductivity as well as inferior ion accessible area, which have resulted in large irreversible capacity, poor rate performance, and fast capacity fading [25, 26]. To deal with these challenges, constructing three-dimensional (3D) carbon superstructures (CSSs) assembled by two-dimensional (2D) hard carbon sheets has been proven to be effective approach for the excellent potassium-ion storage performance [7, 23, 27]. Because these 3D materials can inherit the exceptional properties of their building blocks and acquire certain unconventional advantages, such as electrically conducting network that permits enhanced electrical conductivity or the ion transport during electrochemical reactions, thus leading to improved rate capability and cycling stability [28–30]. Unfortunately, lots of morphology modulation work is just to gather two-dimensional (2D) carbon plates into assembly structure, without interconnected electric conduction path and thus with poor rate performance. Moreover, highly heteroatom-doped 3D carbon materials rich in exposed active facets generally require multistep syntheses and harsh conditions, which are undesirable for practical PIBs. Thus, developing an eco-efficient synthetic method to produce an ideal 3D carbon material with abundant N-doped active facets to enhance its rate performance and long-life storage capability is still a great challenge.

Herein, we propose an eco-efficient synthetic route to establish N-rich carbon superstructures (denoted as NCS) based on 3D morphology evolution, leading to progressively exposing N-rich active facets. The developed route shows several apparent advantages compared with previously reported methods. First, the synthesis process is simple and competitive, without undesirable reagents and/or sophisticated or multistep processes. Second, the spherical morphology assembled by interconnected ultra-thin carbon nanosheets not only offers excellent structural stability, but also leads to beneficial influences on higher bulk density and tap density, which can increase the energy density of the battery. Third, compared with post-treatment of well-defined carbon materials, the exposure of N-rich active facets and N-doping level is regulated by the polymerization process and thus favorable to the optimization of surface-driven electrochemical performance [22, 23]. Benefiting from these merits, the optimized NCS-5 as an anode material for PIB delivers high reversible specific capacity (250 mAh g^−1^ at 200 mA g^−1^ after 300 cycles), outstanding rate capability (190 mAh g^−1^ at 4000 mA g^−1^), and promising cycling performance (205 mAh g^−1^ at 1000 mA g^−1^ after 2000 cycles). To explore the origin of these experimental results, we also conduct detailed DFT calculations to explore the reason for the ultra-high pyridinic/pyrrolic content of the NCS structure.

## Experimental Section

### Synthesis and Structural Characteristics of NCS

**Fig. 1:**
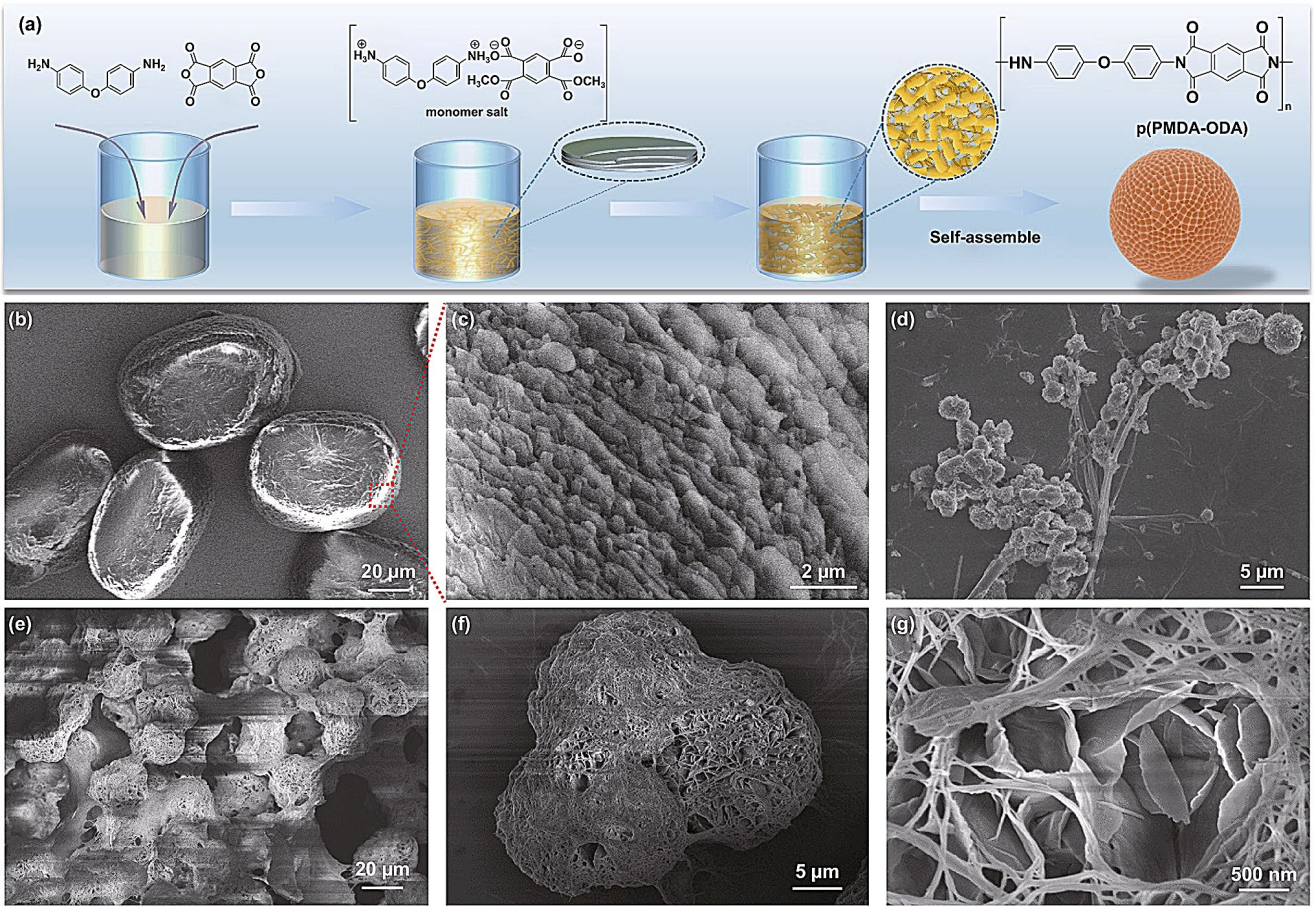
Synthesis of polyimides (PIs). (**a**) Schematic of PI synthesis. (**b, c**) SEM images of [ODA^2+^PMDA^2−^] salts at different magnifications. (**d**) Products obtained by polymerization at 160 °C for 10 min. (**e–g**) Products covered with PVP at different magnifications (polymerization at 160 °C for 2 h)

The typical procedure used to prepare the polyimide (PI) particles is illustrated in Fig. 1a. We used nitrogenous monomers to prepare the monomer salts via salt formation. The monomer salts were synthesized by stirring the diamine 4, 4′-diaminodiphenyl ether (ODA) with an equimolar amount of benzene-1, 2, 4, 5-tetracarboxylic dianhydride (PMDA) in methanol for 2 h at room temperature. The monomer salts [ODA^2+^PMDA^2−^] were isolated as a creamy white powder (Figure S1, Supporting Information) and purified by filtration. Then, dried monomer salts (2.0 g) and polyvinylpyrrolidone (PVP, 0.2 g) were dispersed in ethylene glycol (80 mL). The mixture was polymerized at 160 °C for 2 h with magnetic stirring at 300 rpm under an N_2_ atmosphere. The PI particles were isolated as a brown powder by centrifugation at 9000 rpm for 5 min and then purified by filtration with water three times (Fig. S2). The as-synthesized PIs contained covalently bonded rigid aromatic units in the main chain. This ensured their high structural and thermal stability when subjected to thermal treatment at 600 °C for 1 h in an argon atmosphere to produce NCSs.

### Material Characterization

Sample morphology was characterized by scanning electron microscopy (SEM; Hitachi S4800) and transmission electron microscopy (TEM; JSM-2100 at 200 kV). X-ray diffraction (XRD) patterns of samples were measured by a Bruker D8 Advance X-ray diffractometer using Cu Kα radiation (*λ* = 0.15406 nm). The surface chemical composition of samples was evaluated by X-ray photoelectron spectroscopy (XPS; Escalab 250Xi) using an Al Kα X-ray source operating at 12 kV. Raman spectra were recorded by a WITec Alpha 300R with a laser wavelength of 514 nm. Thermogravimetric analysis was performed using a Shimadzu DTG-60 analyzer.

### Electrochemical Measurements

Working electrodes were fabricated by mixing an NCS, carbon black, and carboxymethyl cellulose sodium salt at a weight ratio of 80:10:10. The mixture was coated uniformly on copper foil using a doctor blade. After drying at 110 °C under vacuum for 12 h, the electrodes were cut into circles with a mass loading of ~ 1.5 mg cm^−2^. Batteries were assembled in an N_2_-filled glovebox. K metal foil used as counter and reference electrodes was separated from the working electrode using a glass microfiber filter (Whatman, Grade GF/D). The electrolyte was 0.8 M KPF_6_ dissolved in ethylene carbonate and propylene carbonate with a 1:1 volume ratio. The as-assembled CR2025-type coin cells were aged for one day before electrochemical property testing.

Galvanostatic discharge/charge tests were performed at various rates in the potential range of (0.01–3.0 V vs. K/K^+^) on a Land CT2001A battery testing system at room temperature. Electrochemical impedance spectroscopy (EIS) and cyclic voltammetry (CV) were conducted on a CHI 660E (Chenhua, China) electrochemical workstation. Energy densities were calculated based on the mass of anode active material.

## Results and Discussion

### Morphology and Structure Analyses of PIs and NCS

Polyimide particles were obtained in a single step via the polycondensation of ODA-PMDA monomer salts using PVP as a steric stabilizer in ethylene glycol. Since the preparation of a monomer salt generates a precursor of ideal stoichiometry that is needed for obtaining high conversion and degrees of polymerization according to Carothers′ Law [31], the polyimide particles grew gradually and stably during the polycondensation process. [ODA^2+^PMDA^2−^] monomer salt has a layer-by-layer nacre-like morphology of polycrystalline crystallites with diameters of 50−100 µm (Fig. 1b, c). Fourier transform infrared analysis (Fig. S3) of the dried monomer salts revealed the coexistence of typical monomer salt modes without any characteristic imide or amide modes. The morphology of p (ODA-PMDA) is strikingly different from the monomer salt and also evolves with polymerization reaction time (*t*_R_): the [ODA^2+^PMDA^2−^] salt rapidly nucleates to form tiny particles, and the particles are stable in dispersion and uniform in size at *t*_R_ = 10 min (Fig. 1d). With increasing *t*_R_, the particles grow into a fascinating morphology: p(ODA-PMDA) presented microflower-like spherical particles composed from 2D nanosheets covered by PVP (Fig. 1e–g) at *t*_R_ = 2 h. By controlling the concentrations of monomer salts, a series of PIs with stepwise changes in morphology were obtained, which are denoted as PI-1, PI-2, PI-3, PI-4, PI-5, and PI-6, corresponding to monomer salt concentrations of 10, 20, 30, 40, 50, and 60 mg mL^−1^, respectively. PI-1, PI-2, PI-3, PI-4, PI-5, and PI-6 were pyrolyzed in an argon atmosphere (600 °C, 2 h) to produce PI-derived carbon superstructures, which are denoted as NCS-1, NCS-2, NCS-3, NCS-4, NCS-5, and NCS-6, respectively.

**Fig. 2:**
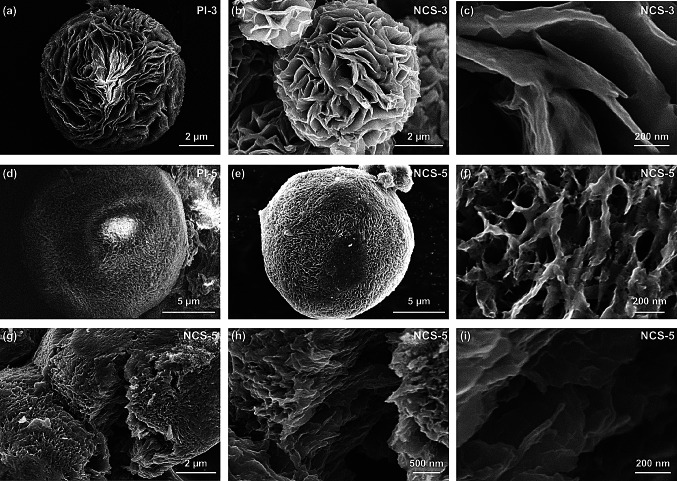
SEM images of (**a**) PI-3, (**b, c**) NCS-3, (**d**) PI-5, and (**e, f**) NCS-5 at different magnifications. (**g–i**) Cross-sectional SEM images of NCS-5 at different magnifications

As shown in Figs. 2, S5 and S6, all the six materials of PIs prepared using different monomer salt concentrations exhibited stepwise changes of their 3D hierarchical architectures, although they all comprised of self-assembled 2D nanosheets. With the increase in the monomer salt concentration, the hydrangea-like PI-3 has more and thinner nanosheets than the PI-1 and PI-2. Moreover, the high concentration of monomer salt endows PI-5 with a spherical sponge-like morphology (Fig. 2d) and gives the resultant NCS-5 with the thinnest and smallest nanosheets (Fig. 2e, f). In order to investigate the internal structure of the sphere, the NCS-5 was violently destroyed for more detailed observations. Cross-sectional SEM images showed that the carbon sheets of NCS-5 were interconnected and curved, integrating into a compact porous 3D architecture (Fig. 2g–i). The porous nanostructure of NCS-5 benefits to ion transfer during electrochemical reactions by effectively shortening the ion diffusion length in the solid phase, which is especially important for electrodes with a high mass loading of active materials. In addition, the interconnected structure of NCS-5 could enable fast electron transfer between different nanosheets, thus improving electrochemical kinetics and corresponding rate performance. It is worth noting that increasing the monomer salt concentration to 60 mg mL^−1^ is unfavorable for the formation of spherical structure (Fig. S6), which against the high bulk density and tap density, and thus reduces the energy density of the battery.

**Fig. 3:**
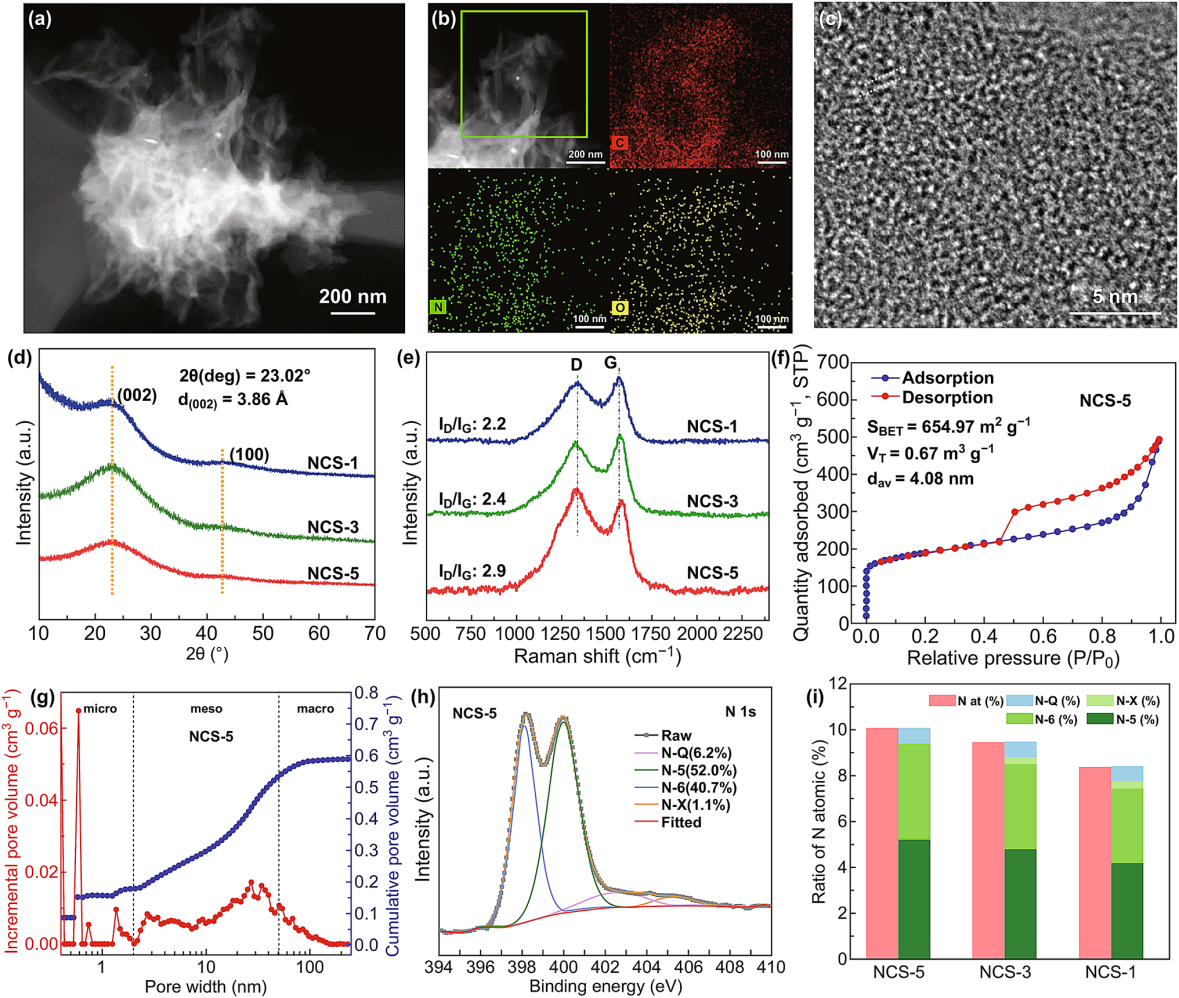
Structural and compositional characterization of selected NCSs. (**a**) HAADF image of NCS-5. (**b**) HAADF image and corresponding EDS elemental mapping of NCS-5. (**c**) HRTEM image of NCS-5. (**d**) XRD patterns of NCSs. (**e**) Raman spectra NCSs. (**f**) Nitrogen adsorption–desorption isotherms of NCS-5. (**g**) Pore size distribution of NCS-5. (**h**) High-resolution N 1s spectra of NCSs. (**i**) Distributions of different types of N-doping in selected NCSs

TEM was used to further investigate the morphology of NCS-5. The high-angle annular dark field (HAADF) image in Fig. 3a shows that the nanosheets of NCS-5 were corrugated and polyporous. Elemental mapping analysis (Fig. 3b) revealed the homogeneous distributions of carbon (C), N, and oxygen (O) in the NCS-5 nanosheets. The high-resolution transmission electron microscopy (HRTEM) image of NCS-5 in Fig. 3c contains turbostratic lattice fringes, indicating that NCS-5 consisted of amorphous carbon. A large interlamellar distance of ~ 3.86 Å at 2*θ* = 23.02° ascribed to the (002) plane of graphite was observed in XRD patterns of NCS-5. The combination of N-doping and turbostratic stacking can facilitate K^+^ insertion into the layered structure of the NCS sheets and accommodate more K^+^ and tolerate the volume expansion.

The Raman spectrum of NCSs, as shown in Fig. 3e, displays two broad peaks, where the D band (~ 1333 cm^−1^) is assigned to the A_1g_ vibration of the C_6_ rings induced by defects, and the G band (~ 1578 cm^−1^) corresponds to the E_2g_ vibration of the *sp*^2^ carbon atoms [32]. The highest intensity ratio of the D band to the G band (*I*_*D*_*/I*_*G*_) of 2.9 indicates that NCS-5 contained a high degree of defects, which benefit for K-adsorption. The *I*_D_*/I*_G_ ratio can be used to calculate the coherence length of the graphenic domains along lateral ab planes (La) according to the empirical formula [33]: *L*_*a*_ (nm) = (2.4 × 10^−10^) *λ*^4^ (*I*_D_*/I*_G_)^−1^, where λ is the laser wavelength [34]. The calculated La of 3.6 nm reveals the extent of the defective and turbostratic structure, which is consistent with the result in HRTEM. The specific surface area and pore-size distributions of NCSs were analyzed using N_2_-adsorption (Figs. 3f, g, S7, and Table S1). All NCSs exhibit type IV isotherms with type H3 hysteresis loops, showing a hierarchical pore distribution of micropores, mesopores, and macropores, which could offer pore channels for ion infiltration and transmission, thereby contributing to fast charging/discharging properties of the NCSs. Besides, the Brunauer–Emmett–Teller (BET) surface areas were calculated to be 679, 652, and 654 m^2^ g^−1^ for NCS-1, NCS-3, and NCS-5, respectively, indicating that morphological change has minimal effect on the resultant surface area in the employed range.

Elemental analysis (EA) and X-ray photoelectron spectroscopy (XPS) were performed to examine the chemical compositions of the NCSs. The EA results revealed that the weight percentage of nitrogen in NCS-1, NCS-3, and NCS-5 was 8.45, 8.88, and 9.12 wt%, respectively, which indicates that the morphology and structure evolution of the NCSs strongly determined the content of N-doping. The high-resolution XPS scanning at N 1 s range can be divided into four characteristic peaks: N-6 (398.0 eV, 40.7%), N-5 ( 400.0 eV, 52.0%), N-Q (402.8 eV, 6.2%), and oxidized pyridine-N (N-X, 404.8 eV, 1.1%), as shown in Fig. 3h. Deconvolution of the N peaks revealed that the total content of N-5 and N-6 in NCS-5 is one of the highest reported values among carbonaceous materials [23, 24, 35–37]. Some reports indicated that the content of active N in N-doped carbon might be the key parameter determining its reactivity. Such N-rich active facets and associated defects could enhance the surface-driven capacity by reversibly binding with the charge carriers and exhibit fast kinetics, as compared with the more inert N-Q [38–41]. As for N-X, which typically represent the oxidized pyridine nitrogen atoms, are incorporated with other N-doped species and usually show enhanced wettability [24]. The high-resolution O 1 s spectra of the NCSs (Fig. S8g–i) exhibited three peaks centered at 531.1 eV (C = O), 532.3 eV (C–OH/C–O–C), and 533.7 eV (COOH). FTIR analysis further demonstrated the high oxygen-containing and nitrogen-containing groups of NCSs (Fig. S9), which is in accordance with the high O content from the EA results and lowest graphitization degree from Raman analysis (*I*_D_/*I*_G_ = 2.9). The presence of O can enhance the wettability of carbon composites, which increases the utilization of their high specific surface area [42, 43].

As previously reported, N heteroatoms can effectively enhance the capacity and rate performance of N-rich carbonaceous anodes. Xu et al. [23] reported that N-6 is energetically favorable for adsorbing K^+^. Yang and co-workers demonstrated that N-5 centers can induce many vacancies and defects, which are reported to increase capacity [27]. Unlike many easy cleavage nitrogen-rich polymers, PI’s high thermal stability and controllable morphology benefit it to transform into carbon materials with a unique morphology. However, the correlation of N-doping species to the morphology of PI was not presented due to the natural difficulty of controlling the N-doping level when dealing with synthesis PI, and the K^+^ storage performance was missing too. Given the advantages of PI-derived carbon, it is of high importance to study the correlation of N-doping species of PI-derived carbon and K-storage performance.

### Electrochemical Performance as PIB Anodes

To investigate the K^+^ storage properties of their NCSs, their electrochemical properties were first examined using CV at a scan rate of 0.1 mV s^−1^. Figure S10a shows the first four consecutive CV curves of an electrode made from NCS-5. The first cathodic cycle contained a large and broad peak at 0.38 V that could be caused by the decomposition of the electrolyte and formation of a solid–electrolyte interphase (SEI) film on the surface of the carbon electrode. Interestingly, the CV curve of NCS-5 showed larger charging currents at lower voltages than those of NCS-3 and NCS-1 (Fig. S10a–c), which indicates the greater possibility of K^+^ intercalation in the thin carbon sheets in NCS-5 than in the other NCSs. Figure S9d shows the potassiation and depotassiation of the NCS-5 electrode in its first cycle at 50 mA g^−1^. The initial irreversible capacity loss of the NCSs electrodes was mainly caused by structural defects and SEI formation, and which was consistent with the CV results.

**Fig. 4:**
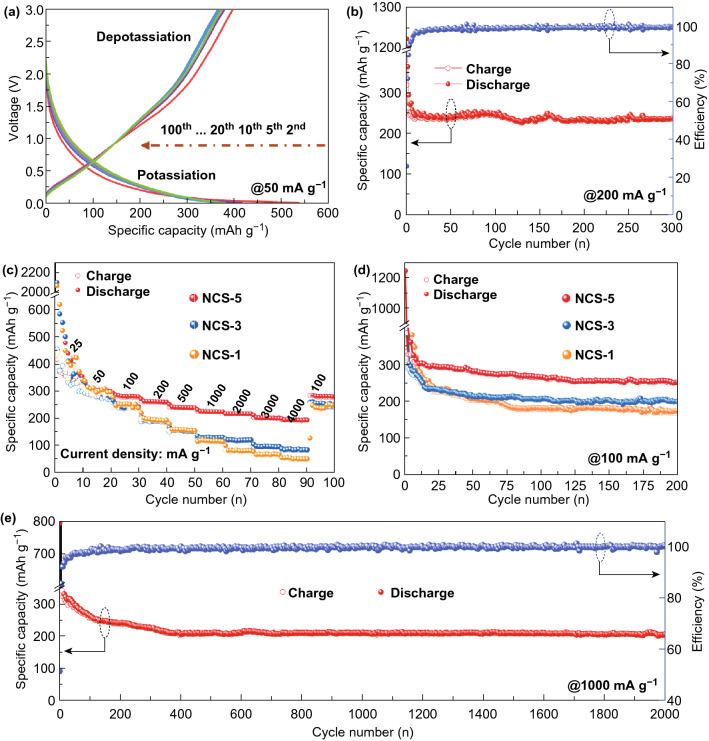
Electrochemical potassium-ion storage performance of NCSs. (**a**) Profiles of the second, fifth, tenth, 20th, 50th, and 100th potassiation and depotassiation cycles of the NCS-5 anode at 50 mA g^−1^. (**b**) Potassiation and depotassiation capacity and Coulombic efficiency of the NCS-5 anode at 200 mA g^−1^. (**c**) Rate capability of the NCSs anodes at current densities from 25 to 4000 mA g^−1^. (**d**) Cycle life of the NCSs anodes measured at 100 mA g^−1^. (**e**) Long-term cycling stability and Coulombic efficiency of the NCS-5 anode at 1000 mA g^−1^

Figure 4a shows typical charge/discharge curves of the NCS-5 electrode at 50 mA g^−1^. Stable potassiation and depotassiation capacities for the NCS-5 electrode were observed after ten cycles. Figure 4b depicts the cycling performance and Coulombic efficiency of the NCS-5 electrode at 200 mA g^−1^ over 300 cycles. A high sustained potassiation capacity of 250 mAh g^−1^ after 300 cycles was observed, and the Coulombic efficiency was as high as 99.9%, illustrating the excellent cycling stability of the NCS-5 electrode for PIBs. The NCS-5 electrode also exhibited the highest stability and reversibility of the tested anodes at different current densities (Fig. 4c). The electrochemical performance of NCS-5 was higher than that of the other NCS anodes from 25 to 4000 mA g^−1^; the NCS-5 anode displayed reversible capacities of 302, 280, 256, 231, 220, 208, 199, and 192 mAh g^−1^ at 50, 100, 200, 500, 1000, 2000, 3000, and 4000 mA g^−1^, respectively. Importantly, after 90 successive cycles at different current densities, the capacity of the NCS-5 anode was 279.5 mAh g^−1^ when the current density was reset to 100 mA g^−1^, showing a great merit for an abuse tolerance with varied current densities. The cycling stabilities of NCS-5, NCS-3, and NCS-1 anodes at 100 mA g^−1^ are compared in Fig. 4d. The durability of the NCS-5 electrode was further revealed by its reversible capacity of 205 mAh g^−1^ at 1000 mA g^−1^ for over 2000 cycles (Fig. 4e), representing competitive performances among all reported carbonaceous PIB anodes.

Based on these data, we conclude that: (1) enhancing the exposure of N-rich active facets is a favorable strategy to increase the specific capacities for carbonaceous materials, as shown by the performance comparison between the NCS-1 and NCS-5 samples, and (2) the synergistic effect of the 3D interconnected superstructures and the nitrogen doping endows the carbonaceous materials with not only increased potassium-ion storage capabilities but also superior rate and cycling performance. The comparison of potassium-ion storage performances among our work and the relative literatures are displayed in Table S2, which can further indicate that the NCS-5 anodes exhibit excellent cycle stability for PIBs.

**Fig. 5:**
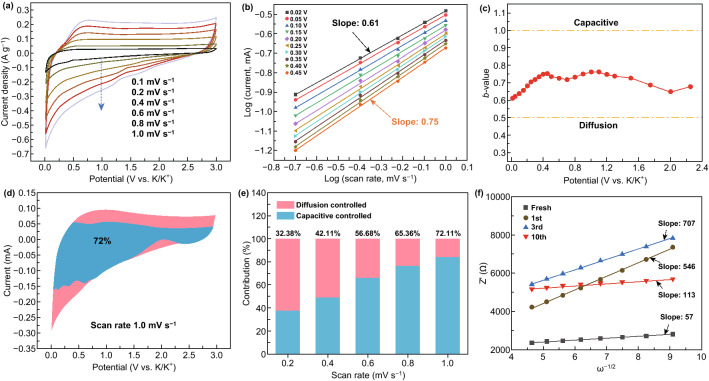
(**a**) CV curves of the NCS-5 anode at different scan rates in the voltage range of 0.01–3.0 V (vs. K^+^/K). (**b**) Derived relationship between peak current and scan rate in logarithmic format. (**c**) *b*-values plotted as a function of potential for the cathodic scan (K^+^ intercalation). (**d**) Separation of the capacitive and diffusion currents in the NCS-5 anode at a scan rate of 1.0 mV s^−1^. (**e**) Normalized contributions of capacitance and diffusion at different scan rates. (**f**) Relationship between Z′ and ω^−1/2^ in the low-frequency region

Because of the gratifying performance of the NCS-5 anode, it is meaningful to qualitatively and quantitatively study the contributions of diffusion-controlled intercalation and surface-induced capacity to the potassiation and depotassiation processes of this anode [2]. To further evaluate the electrochemical behavior and kinetics of NCS-5, CV was carried out in the potential range from 0.01 to 3.0 V at scan rates from 0.1 to 1.0 mV s^−1^. The quasi-rectangular shape of the CV curves in the high potential region (1–3 V) resembled the behavior of a double-layer capacitor, which indicates that the large specific surface area of NCS-5 is conducive to non-Faradaic reversible adsorption (Fig. 5a). In addition, broad potassiation/depotassiation peaks were maintained at high scan rates and the peak shift toward higher potential can be clearly seen in the CV curves. The distortion of the rectangular shape could be ascribed to a surface-driven process, where K^+^ binds to various surface and near-surface heteroatom moieties and graphene defects. Because the N-doped active sites possess a distribution of adsorption energies, the plots of voltage against capacity have irregular slopes. Similar behavior was observed for reported N-doped hard carbon materials used in Na^+^ and Li^+^ storage systems [44–46].

Regarding the specific contribution ratio of the two mechanisms to the performance of the NCS-5 anode, the power-law relationship between the scan rate (*ν*) and current *(i*), *i* = *aν*^*b*^, was established by analyzing the CV data obtained at different sweep rates. Parameter *b* was determined by plotting log(*i*) against log(*ν*) (Fig. 5b). Generally, a *b*-value approaching 0.5 indicates an ideal diffusion-controlled process, and a *b*-value close to 1.0 indicates a surface capacitive-controlled process [47, 48]. Our calculation indicated that the *b*-value of the NCS-5 anode was between 0.6 and 0.8, suggesting a mixture of mechanisms of K^+^ charge storage occurred in this electrode (Fig. 5c). To quantitatively separate the contribution of surface capacitive-controlled and diffusion-controlled elements, the relationship between current response and scan rate can be described by Dunn’s method [49]. Figure 5d shows typical CV profiles at a scan rate of 1.0 mV s^−1^ for the current from the surface capacitive-controlled process (blue area) compared with the total current. A surface process contribution of 72.11% was obtained for the NCS-5 anode, confirming that most charge storage at 1.0 mV s^−1^ is associated with capacitive-controlled processes. The capacitive charge contribution for the NCS-5 electrode at scan rates from 0.2 to 1.0 mV s^−1^ is displayed in Fig. 5e, in which the diffusion- and capacitive-controlled regions are shown in red and blue, respectively. The proportion of the capacitive contribution gradually increased with the scan rate and finally reached the maximum value of 72.11% at 1.0 mV s^−1^, demonstrating that the surface capacitive-controlled process is the main K^+^ storage mechanism at a high scan rate.

To further understand the high rate capability of NCS-5, the charge transfer behavior of K^+^ was studied by EIS (Fig. S11). Nyquist plots of the potassium half-cell showed a large semicircle, indicating a high total impedance. It is known that the impedance in a potassium half-cell is the total impedance of the working electrode and potassium metal electrode at the same frequencies. In view of this, the impedance properties of the working electrode would be masked by the large impedance of the potassium metal electrode because of its high chemical activity [50].

The diffusion coefficient (*D*) of K^+^ was calculated using Eqs. 1–3 [51]:

1 $$\omega \, = \,{\text{2}}\pi f$$

2 $${\text{Zre}}\, = \,R\, + \,\sigma \omega ^{{ - {\text{1}}/{\text{2}}}}$$

3 $$D\, = \,0.{\text{5}}R^{{\text{2}}} T^{{\text{2}}} /A^{{\text{2}}} n^{{\text{4}}} F^{{\text{4}}} C^{{\text{2}}} \sigma ^{{\text{2}}}$$

where *R* is the gas constant (8.314 J mol^−1^ K^−1^), *T* is the absolute temperature (293.15 K), *A* is the surface area of the electrodes (1.13 cm^2^), *F* is the Faraday constant, *C* is the molar concentration of K^+^ (0.8 M), *σ* is the Warburg coefficient, and *n* is the number of electrons transferred per molecule [35, 52]. *σ* was determined from the slope of a plot of *Z*′ versus ^−1/2^, which exhibited a gradually decreasing trend and a low value of 113 after the first ten cycles, as shown in Fig. 5f. *D* decreased from 1.18 × 10^−12^ cm^2^ s^−1^ (fresh) to 3.58 × 10^013^ cm^2^ s^−1^ (after one charge–discharge cycle) and then improved to 1.07 × 10^−12^ cm^2^ s^−1^ (after 10 charge–discharge cycles), demonstrating the excellent structural stability and fast diffusion of the NCS-5 anode. *D* exhibited an obvious decrease between the first and third charge–discharge cycles, which was ascribed to the large polarization and impedance in the potassium half-cell caused by the high chemical activity of potassium [50].

As mentioned above, the exceptional electrochemical properties of the as-prepared NCS-5 anode could be attributed to three reasons. First, the surface-driven K^+^ storage mainly occurred in the surface and near-surface regions and proceeded with fast kinetics, making it beneficial for high rate capability. The abundant N-rich active facets and associated defects of NCS-5 enhanced the surface-driven capacity by reversibly binding with the charge carriers and exhibit fast kinetics. Second, the ultra-thin and small carbon sheets and expanded interlayer spacing effectively shortened the K^+^ diffusion distance in the solid phase. Third, the interconnected 3D structure of NCS-5 is an electrically conducting network that permits fast electron transfer between the different sheets, thus improving electrochemical kinetics to ensure superior rate performance.

### Formation Mechanism of High N-5/N-6 Content

**Fig. 6:**
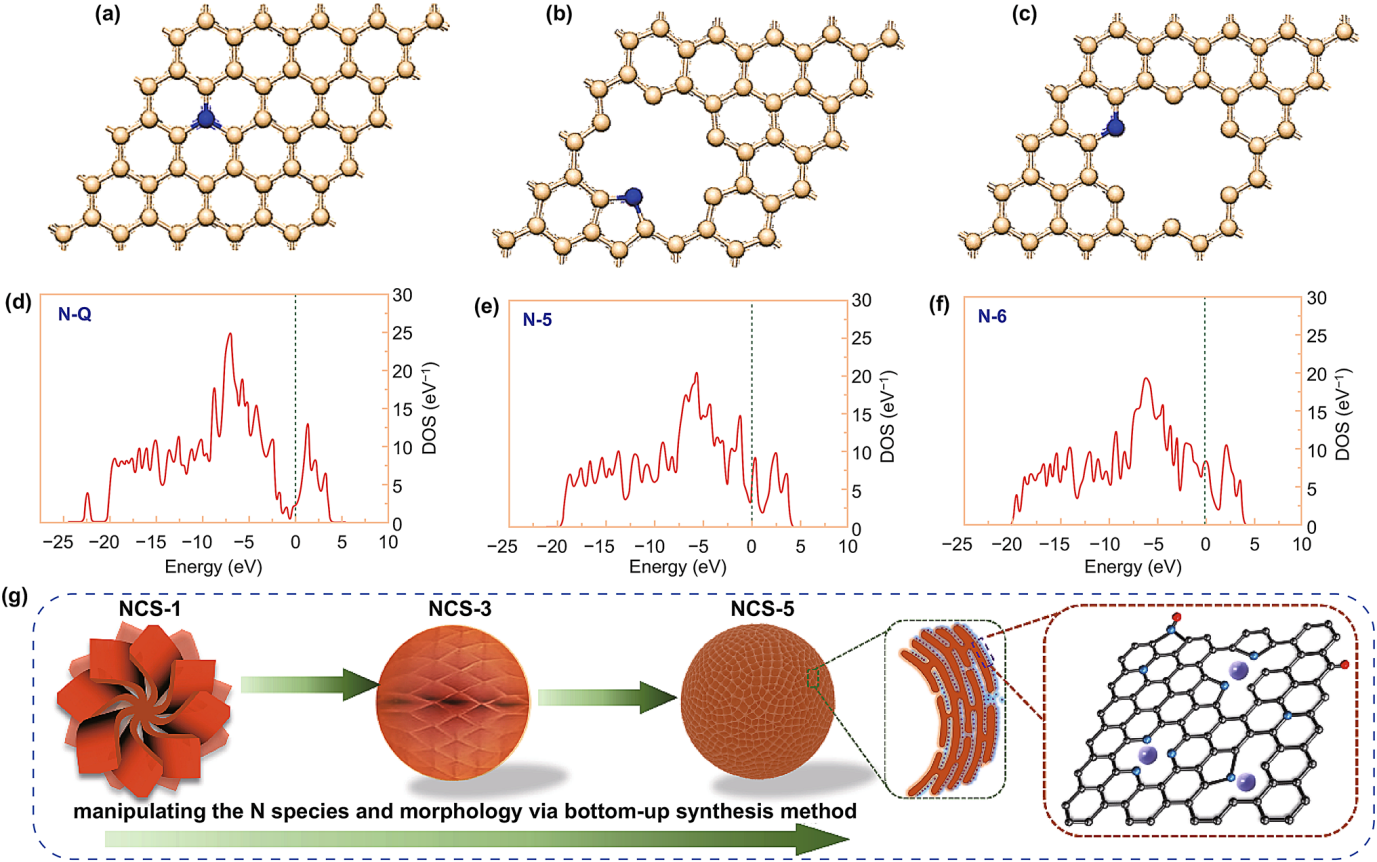
Theoretical simulations of the formation mechanism of N-doped structures. Top views of the configurations of (**a**) N-5, (**b**) N-6, and (**c**) N-Q. Corresponding projected density of states for (**d**) N-5, (**e**) N-6, and (**f**) N-Q. (**g**) Schematic illustration of the hierarchical structure evolution of NCSs

According to the above results, it is concluded that high N-5/N-6 content of NCS-5 significant benefits for charge storage under high current density. Thus, to deeply understand the relationship between N-species contents and molecular structure, the formation energies of three N-doped structures were calculated using DFT [53]. As shown in Fig. S12, the corresponding undoped and vacancy graphene models, i.e., C-5, C-6, and pure graphene (P–C) were established to clarify the effects of doping and defect on the substitution of heteroatoms into monolayer graphene. As revealed from a formation energy calculation (Note S9, Supporting Information), the formation energies of C-5 and C-6 configurations were 17.12 and 18.08 eV, respectively. The positive values suggested that the formation of defects requires high energy. The formation energies of N-5 and N-6 were − 1.78 and − 1.71 eV, respectively, which were much lower than that of N-Q (0.856 eV), suggesting that N-5 and N-6 form readily in graphene vacancies. During the carbonization process, PI precursors undergo a series of complicated chemical action like crosslinking, aromatization, and graphitization, leading to the formation of the defect sites and thus the N-doped sites. Additionally, as evidenced by SEM image (Fig. 2), PI-5 embraces more exposed surfaces area than others PI precursors, which absolutely provide more exposed defects for capturing nitrogen atoms that escaped from the precursor during heat treatment process. Based on above discussions, it is reasonable to say that the high N-doping level of NCS-5 is attributed to the optimized assembly structure via the bottom-up synthesis method. In contrast, because of the stable sp^2^ conjugated structure of well-defined graphene and carbon nanotubes, only a small amount of N atom (1–3 wt%) can be doped into the honeycomb lattice [24]. Besides, the high DOS intensity near the Fermi level (Figs. 6d–f and S11), N-5 and N-6 structures are expected to be more activity as compared with NQ one. Guo et al. have calculated the binding energy of the K ions at different N-doping sites, demonstrating that pyridinic-N and pyrrolic-N are in favor of K ion adsorption [2, 23]. Therefore, vacancy defects together with N-doping could contribute to K ion adsorption and thus enhance surface-controlled capacitive of NCS-5, as previously reported.

Thus, the above calculation results are well consistent with the previous experimental outcomes and strongly certificate the rationality of our manipulating the N species and morphology via the bottom-up synthesis method. To be more intuitional and based on our findings, as illustrated in Fig. 6g, the enhanced exposure of N-rich active facets via hierarchical structure evolution is revealed. The decrease in both of the thickness and size with the increase in monomer salt concentration will lead to the exposure of active facets of nanosheets. Furthermore, the N-rich active facets combined with the robust 3D structure can not only lower the K^+^ diffusion energy barrier, but also provide more stable active facets for capacitive K^+^ storage.

## Conclusions

A series of carbon superstructures with progressively exposed N-doped facets were synthesized by a mild process. Analysis of K^+^ storage performance revealed that NCS-5 demonstrated the highest rate and cycling performance of the investigated anodes and was competitive with other reported carbon-based electrode materials. DFT calculations demonstrated the formation mechanism of pyrrolic and pyridinic-N in the NCSs, which provided evidence for the correlation of high N-doping level of NCS-5 and optimized assembly structure via bottom-up synthesis method. Our results showed that the combination of optimized 3D carbon superstructures with a high content of electrochemical active N species led to improved energy storage by increasing the surface-driven capacitive energy storage, which enhanced the capacity, rate, and cycling performance of the NCS anode active materials. These conclusions are applicable to many other carbonaceous electrode materials, providing an approach to improve structure design, thus leading to high rate capability and stable metal-ion storage.

## Electronic supplementary material

Below is the link to the electronic supplementary material.

Supplementary material 1 (DOCX 13095 kb)
