# Supplementary material for: Enhanced Potassium-Ion Storage of the 3D Carbon Superstructure by Manipulating the Nitrogen-Doped Species and Morphology
Source: Nanomicro Lett. 2020 Oct 27;13:1. doi: 10.1007/s40820-020-00525-y (PMC8187550; doi:10.1007/s40820-020-00525-y)
Supplement: Supplementary file 1 — Supplementary material 1 (DOCX 13095 kb) [file 40820_2020_525_MOESM1_ESM.docx]

**Supplementary Figures and Table**

**Enhanced Potassium-ion Storage of the 3D Carbon Superstructure by Manipulating the N Species and Morphology via Bottom-up Synthesis Method**

Yanhua Li^1, 2^, Kui Xiao^2^, Cong Huang^1^，Jin Wang^3^, Ming Gao^1^, Aiping Hu^1^, Qunli Tang^1^, Binbin Fan^1^, Yali Xu^1^, Xiaohua Chen^1, *^

^1^College of Materials Science and Engineering, Hunan University, Changsha 410082, China.

^2^College of Materials and Chemistry Engineering, Hunan Institute of Technology, Hengyang 421002, China.

^3^Zhuzhou Times New Material Technology Co., LTD, Zhuzhou 412007, China.

* Xiaohua Chen, E-mail: [xiaohuachen@hnu.edu.cn](mailto:xiaohuachen@hnu.edu.cn)

Content

[Note 1 Photograph of monomer salts 2](#_Toc40177339)

[Note 2 Photograph of PI particles 2](#_Toc40177340)

[Note 3 FT-IR and TGA analysis 2](#_Toc40177341)

[Note 4 SEM images 4](#_Toc40177342)

[Note 5 Nitrogen adsorption-desorption isotherms of NCSs 5](#_Toc40177343)

[Note 6 XPS and high-resolution XPS spectra of NCSs 6](#_Toc40177344)

[Note 7 Characteristics (XPS and BET surface area) of NCSs 6](#_Toc40177345)

[Note 8 Electrochemical performance of NCSs as PIB anodes. 7](#_Toc40177346)

[Note 9 Formation energies calculation of the three N-doped carbon via DFT 8](#_Toc40177347)

## Note 1 Photograph of monomer salts

**
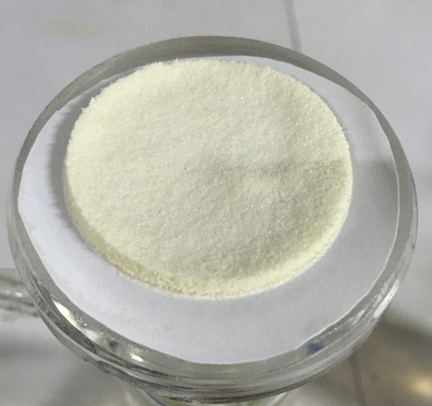
**

Fig. S1. Photograph of monomer salts

## Note 2 Photograph of PI particles


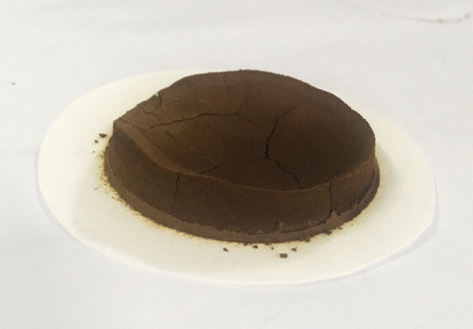


Fig. S2. Photograph of PIs.

## Note 3 FT-IR and TGA analysis

FT-IR spectra were recorded on a Bruker Tensor 27. Resolution was set to 2-4 cm^-1^, and spectra were recorded from 4000 to 600 cm^-1^.


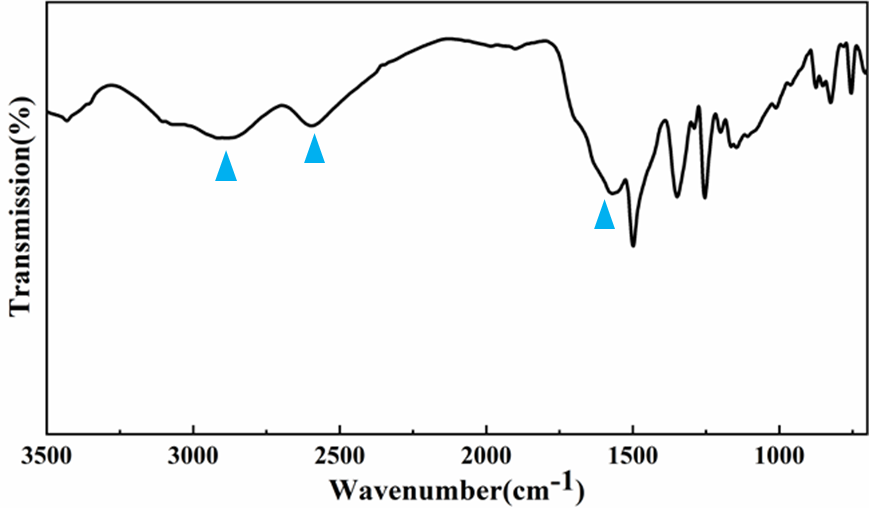


Fig. S3. FT-IR spectra of monomer salt [ODA^2+^PMDA^2-^]. : typical monomer salt modes, ṽ_as_ ( Ar-NH^3+^) ≈ 2840 cm^-1^, ṽ_s_ (Ar-NH^3+^) ≈ 2580 cm^-1^, ṽ_s_(C=O, Ar-COO-) ≈ 1570 cm^-1^.





Fig. S4. TGA curve of PI-5 tested in N_2_ atmosphere.

## Note 4 SEM images


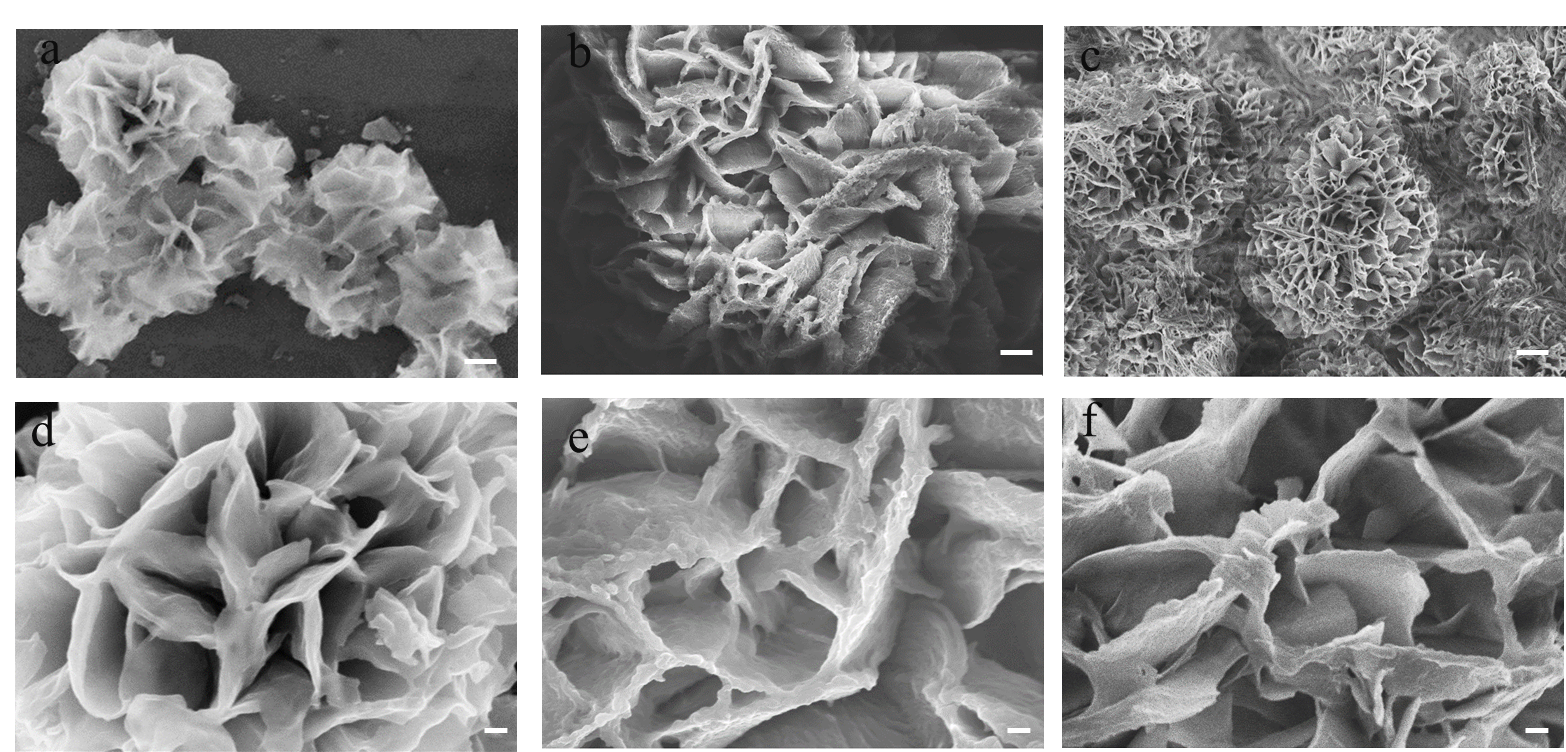


Fig. S5. SEM images of (a) PI-1, (b) PI-2, (c) PI-4, (d) NCS-1, (e) NCS-2, (f) NCS-4. Scale bar: (a)- (c) 2 µm, (d)-(e) 200 nm.


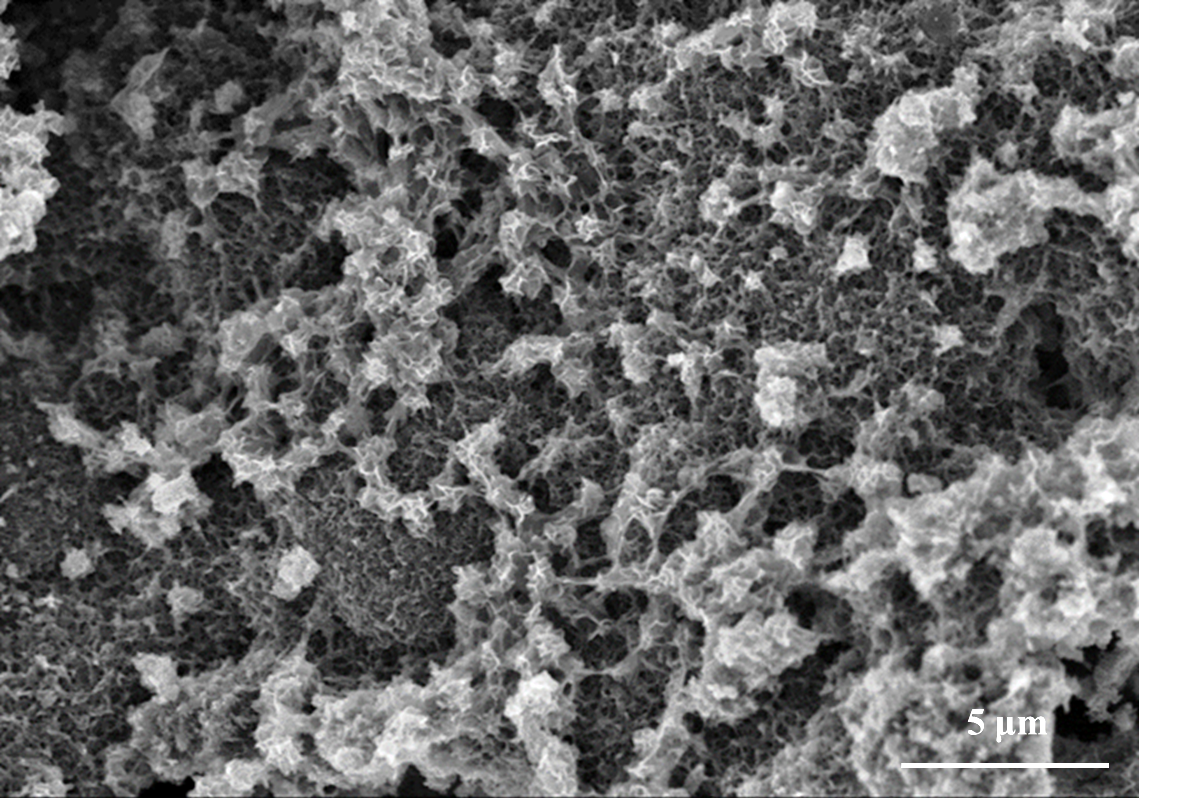


Fig. S6. SEM images of PI-6

## Note 5 Nitrogen adsorption-desorption isotherms of NCSs


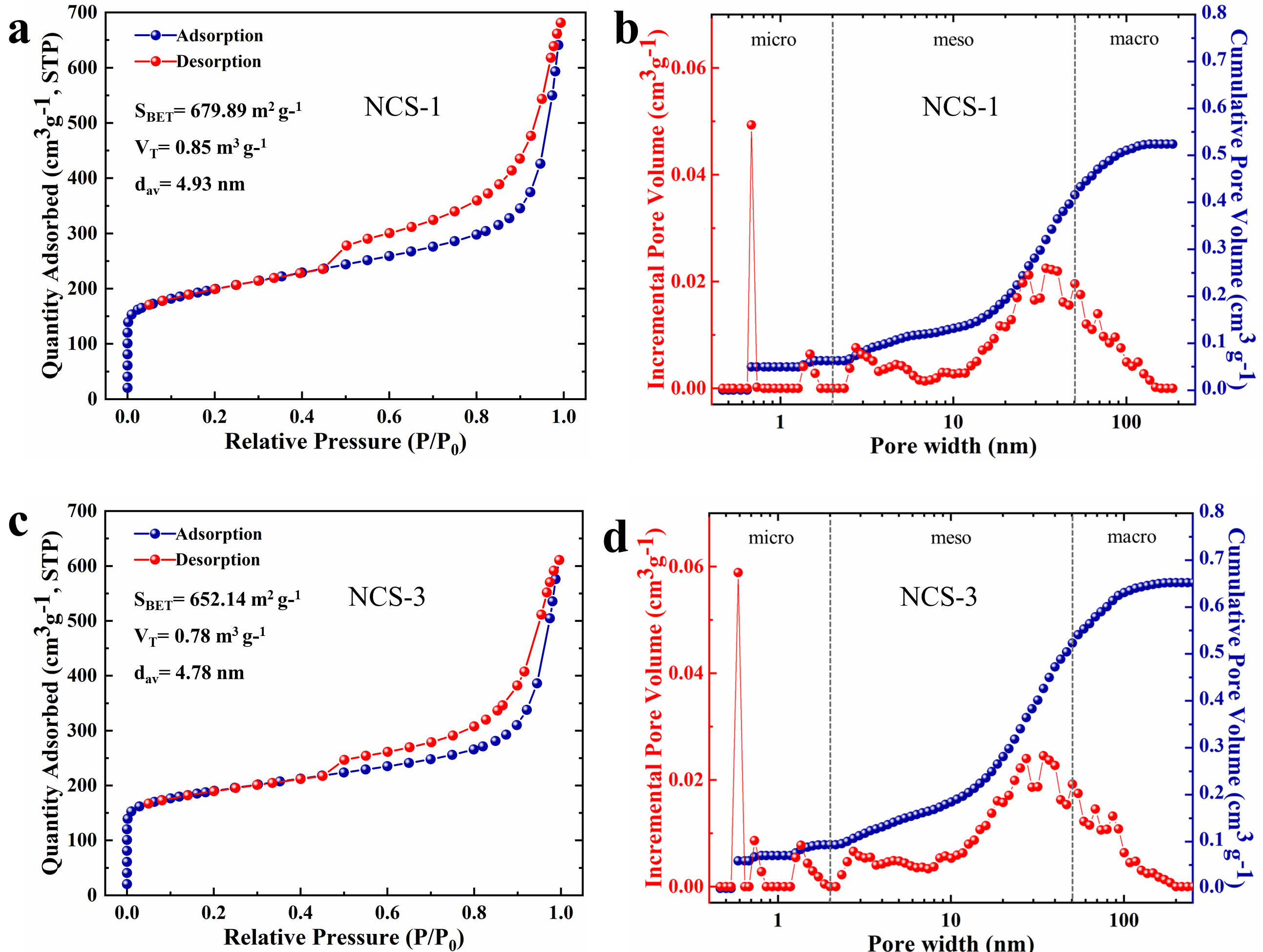


Fig. S7. a) Nitrogen adsorption-desorption isotherms of the NCS-1. b) Pore size distribution of the NCS-1. c) Nitrogen adsorption-desorption isotherms of the NCS-3. d) Pore size distribution of the NCS-3.

## Note 6 XPS and high-resolution XPS spectra of NCSs


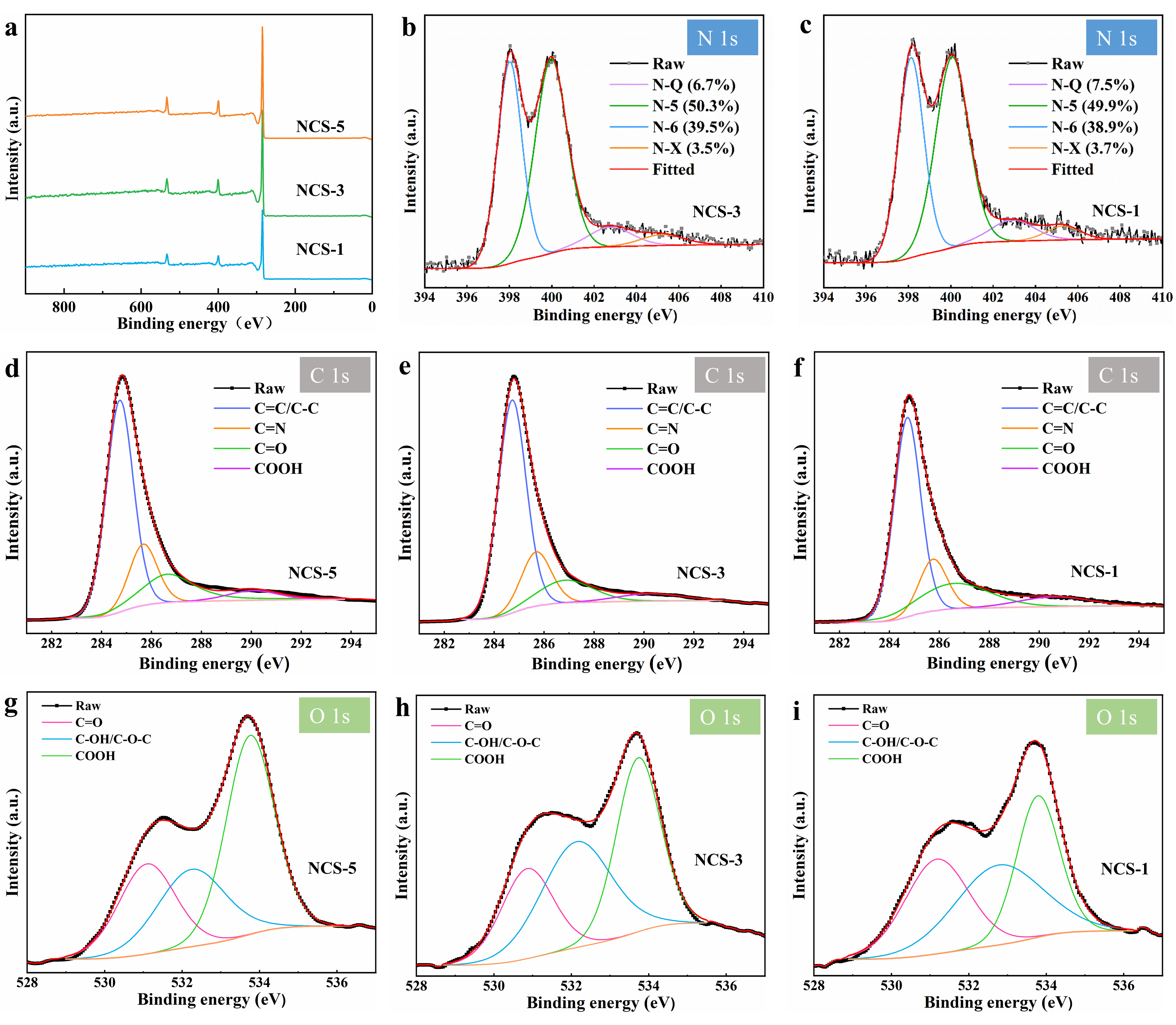


Fig. S8. XPS survey spectra. a) Survey spectrum of XPS of NCSs. N 1s high-resolution XPS spectra of NCSs: b) NCS-3, c) NCS-1. C 1s high-resolution XPS spectra of NCSs: d) NCS-5, e) NCS-3, d) NCS-1. O 1s high-resolution XPS spectra of NCSs: g) NCS-5, h) NCS-3, e) NCS-1.

## Note 7 Characteristics of NCSs

Table S1 Content of element by EA and N-doped type by XPS

| Sample | C (%) | N (%) | H (%) | O (%) | N type (%) | | | |
| --- | --- | --- | --- | --- | --- | --- | --- | --- |
|  |  |  |  |  | N-5 | N-6 | N-X | N-Q |
| NCS-5 | 76.18 | 9.12 | 3.22 | 11.48 | 52.0 | 40.7 | 1.1 | 6.2 |
| NCS-3 | 76.59 | 8.88 | 3.68 | 10.85 | 50.6 | 39.2 | 3.5 | 6.7 |
| NCS-1 | 76.89 | 8.45 | 3.91 | 10.75 | 50.1 | 38.9 | 3.7 | 7.5 |

Fig. S9 presents the FTIR spectrum of PI and NCSs. The bands of PI at 1780, 1720 cm^-1^ are attributed to the stretching and bending vibration of C=O, the peak at 1381 cm^-1^ belongs to the stretching vibration of C-N-C. The other peaks of PI at 1502 and 1248 cm^-1^ are assigned to the C-C stretching of benzene ring and the C-O-C stretching, respectively [1]. Due to the high thermal stability of PI, the weak characteristic peak of the C=O and C-H bond remained in the NCSs after 600 ℃ pyrolysis, as shown in the FTIR spectrum. The emerging peaks of NCSs at 1500–1610 cm^-1^ and 1000–1320 cm^-1^ are assigned to the stretching vibration of the C=C bond and C-N bond derived from the pyrolysis [2]. FTIR results indicate the high oxygen-containing and nitrogen-containing groups of NCSs, which is in accordance with the high O content from EA result and lowest graphitization degree from Raman analysis (I_D_/I_G_=2.9).


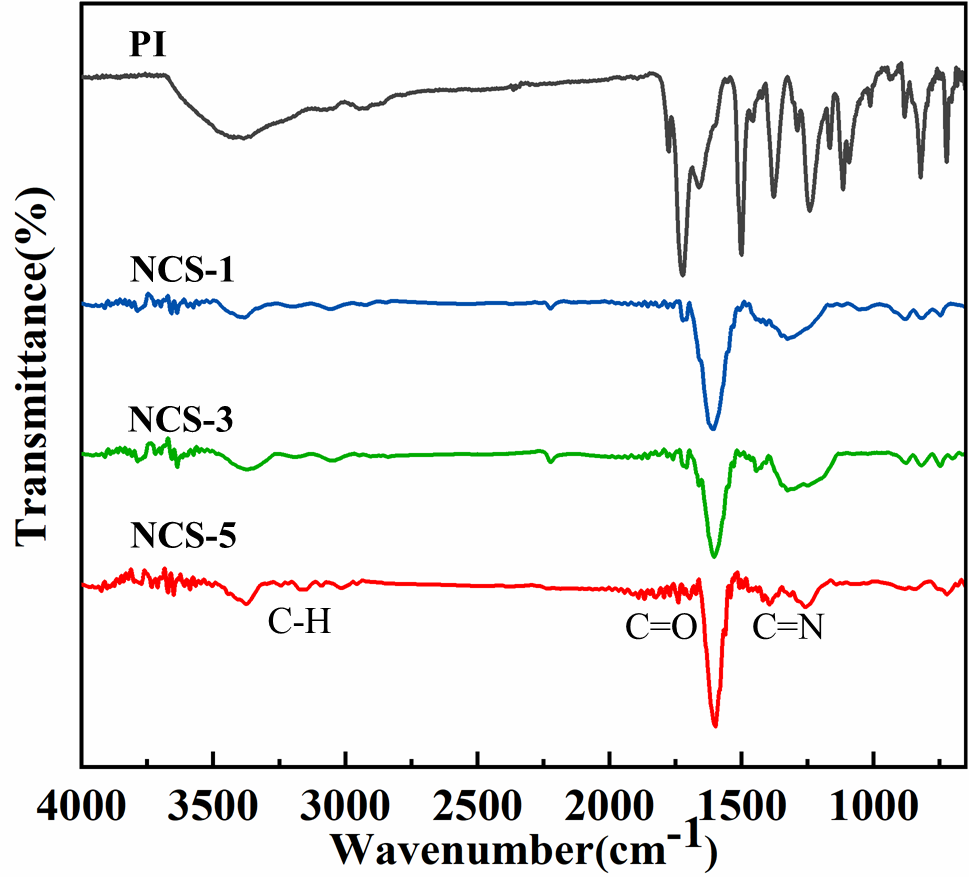


Fig. S9. FI-IR spectrum of PI and NCSs

## Note 8 Electrochemical performance of NCSs as PIB anodes.


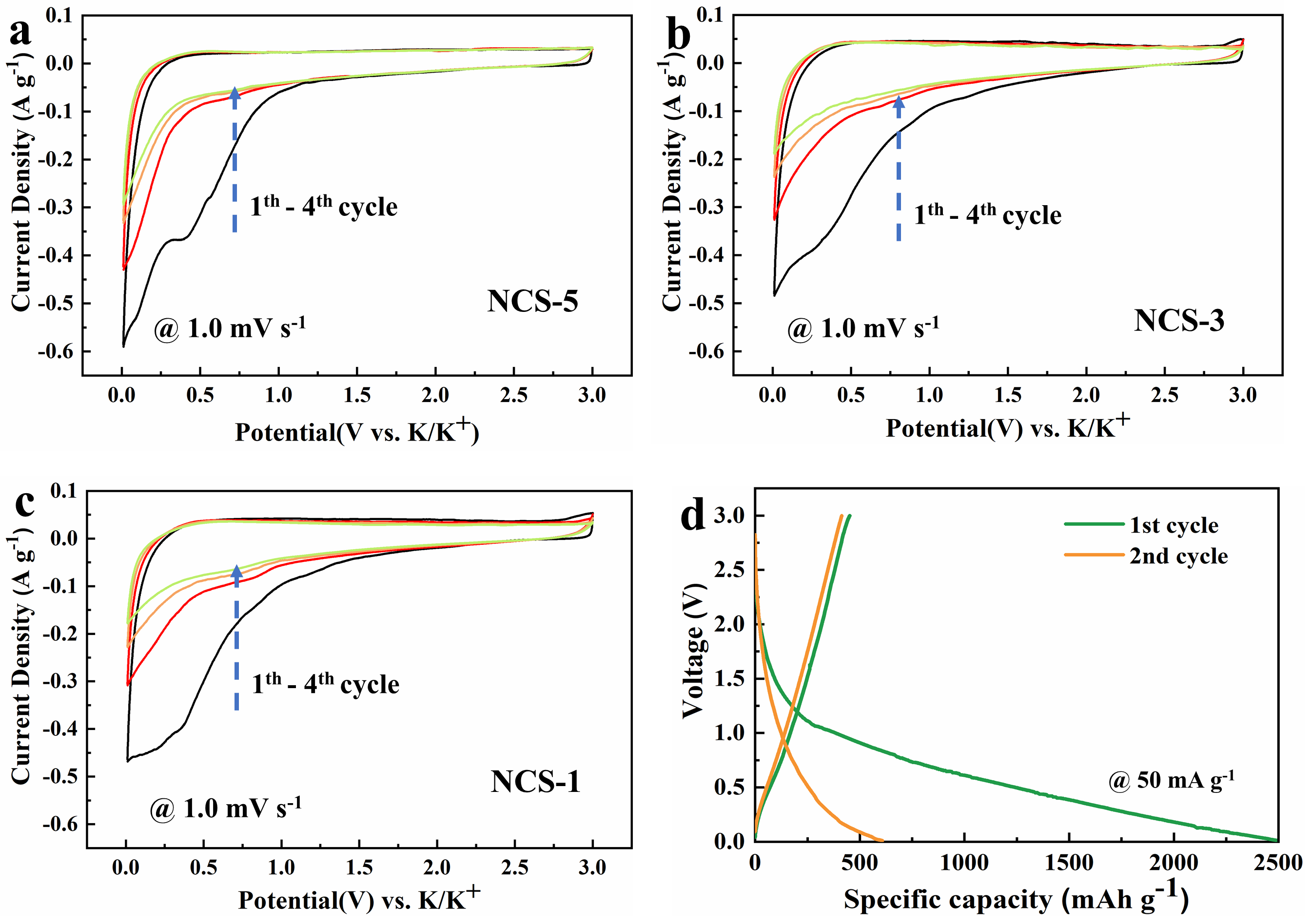


Fig. S10. Electrochemical performance of NCSs as PIB anodes in half cells. a-c) Cyclic voltammetry (CV) of the NCSs electrode for PIBs between 0.01 V and 3.0 V with a scan rate of 0.1 mV/s. d) Potassiation and depotassiation profiles of the NCS-5 electrode for the first cycle at 50 mA g^-1^.





Fig. S11 EIS analysis of NCS-5.

Table S2 Comparison of the potassium-ion storage performances of the NCS-5 anode and carbon anodes published in the relative literatures.

| Materials | Sample  labels | ICE (%)  @ 50 mA g^-1^ | Reversible capacities  mA h g^-1^  @ mA g^-1^ | Cyclability (capacity retention)  mA h g^-1^ @ mA g^-1^@ cycle number | Refs |
| --- | --- | --- | --- | --- | --- |
| N doped 3D carbon superstructure | NCS-5 | 27.8 | 302  @ 50 | 205  @ 1000 @ 2000 | This work |
| graphitic carbon nanocage | CNC | 40  @ 0.2 C | 195  @ 0.2 C | - | [3] |
| hard–soft composite carbon | HCS-SC | 67  @ 0.1 C | 230  @ 0.5 C | 200  @ 1C @ 200 | [4] |
| N/O dual-doped hard Carbon | NOHPHC | 25 | 365  @ 25 | 118  @ 3000 @ 4000 | [5] |
| N doped carbon nanofibers | NCNF-650 | 49 | 248  @ 25 | 146  @ 2000 @ 4000 | [6] |
| N/O dual-doped carbon network | NOCN | 47 | 464.9  @ 50 | 160  @ 5000 @ 4000 | [7] |
| D-doped hierarchical  porous carbon | N-HPC | - | 292  @ 100 | 157  @2000@ 12000 | [8] |
| S-doped RGO sponges | S-RGO-600 | 65 | 361  @ 50 | 229  @1000 @500 | [9] |
| S/O codoped hard carbon | PCMs | 61.7 | 226.6  @ 50 | 108.4  @ 1000 @ 2000 | [10] |
| Phosphorus and oxygen dual-doped graphene | PODG | 22.6 | 474  @ 50 | 160  @ 2000 @ 600 | [11] |
| few-layer F-doped graphene foam | FFGF | 41.2 | 326.1  @ 50 | 165.9  @ 500 @ 200 | [12] |
| Phosphorus doped N-rich honeycomb-like carbon | PNHC | 56.9 | 419.3  @ 100 | 270.4  @ 1000 @ 2000 | [13] |
| free-standing nitrogen-doped carbon nanotube | NCSCNT | 14.2 | 323  @ 20 | 236  @ 20 @100 | [14] |

RGO: reduced graphene oxide; ICE: initial Coulombic efficiency.

“-” stands for unknown value.

1 C = 279 mA g^-1^

## Note 9 Formation energies calculation of the three N-doped carbon via DFT

Calculations were based on the density functional theory (DFT) using the generalized gradient approximation [15] for the exchange–correlation potential prescribed by Perdew–Burke–Ernzerhof, which was implemented in DMol^3^ package [16]. Allelectron calculations were employed with the double numerical basis sets plus polarization functional (DNP). A supercell (5×5) with the periodic boundary conditions on the x–y plane was employed. The vacuum space was set with 20 Å in the z direction to avoid the interactions between periodic images. Parameter settings wee set by the previously reported method with the optimization results [17].

The formation energies of vacancy graphene and vacancy doped-graphene was calculated according to the following definition:

$\Delta E_{1}=E_{vacancy}-E_{pure}+E_{C}$ (1)

$\Delta E_{2}=E_{doped}-E_{vacancy}-E_{N}+E_{C}$ (2)

$\Delta E_{3}=E_{doped}-E_{pure}-E_{N}+E_{C}$ (3)

where $E_{vacancy}$ is the total energy of the vacancy graphene, $E_{doped}$ is the total energy of the doped graphene, $E_{pure}$ is the total energy of the pure graphene, and$E_{C}$ is the total energy from C atom calculated from the corresponding pure/vacancy graphene, $E_{N}$ was obtained from N in the gas phase (N_2_ molecule). All these energies are always taken from simulations using the same basis set.


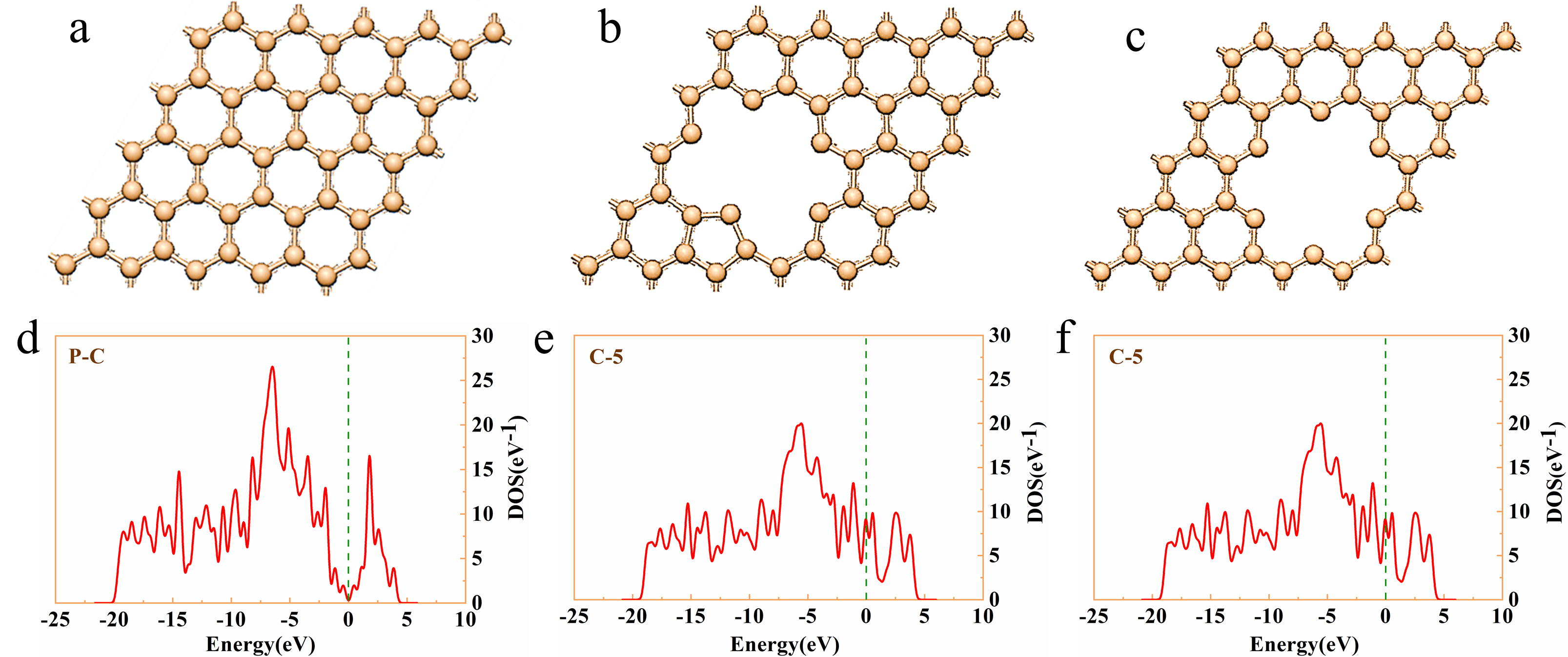


Fig. S12. Top views of the configurations of pure graphene P-C (a), vacancy graphene for C-5 (b), and vacancy graphene for C-6 (c). The density of states (DOS) for pristine C (d), vacancy graphene for C-5 (e), and vacancy graphene for C-6 (f).

**Supplementary References**

1. S. Zeng, L. Guo, F. Cui, Z. Gao, J. Zhou, J. Shi. Template-free synthesis of crystalline polyimide spheres with radiate branches. Mater Lett. **64**(5), 625-627 (2010). [https://doi.org/10.1016/j.matlet.2009.12.024](https://doi.org/https://doi.org/10.1016/j.matlet.2009.12.024)

2. Q. Wu, J. Liu, C. Yuan, Q. Li, H.-g. Wang. Nitrogen-doped 3d flower-like carbon materials derived from polyimide as high-performance anode materials for lithium-ion batteries. Appl Surf Sci. **425**, 1082-1088 (2017). [https://doi.org/10.1016/j.apsusc.2017.07.118](https://doi.org/https://doi.org/10.1016/j.apsusc.2017.07.118)

3. B. Cao, Q. Zhang, H. Liu, B. Xu, S. Zhang, T. Zhou, J. Mao, W. K. Pang, Z. Guo, A. Li, J. Zhou, X. Chen, H. Song. Graphitic carbon nanocage as a stable and high power anode for potassium-ion batteries. Advanced Energy Materials. **8**(25), 1801149 (2018). <https://doi.org/10.1002/aenm.201801149>

4. Z. Jian, S. Hwang, Z. Li, A. S. Hernandez, X. Wang, Z. Xing, D. Su, X. Ji. Hard–soft composite carbon as a long-cycling and high-rate anode for potassium-ion batteries. Adv Funct Mater. **27**(26), 1700324 (2017). <https://doi.org/10.1002/adfm.201700324>

5. J. Yang, Z. Ju, Y. Jiang, Z. Xing, B. Xi, J. Feng, S. Xiong. Enhanced capacity and rate capability of nitrogen/oxygen dual-doped hard carbon in capacitive potassium-ion storage. Adv Mater. **30**(4), 1700104 (2018). <https://doi.org/10.1002/adma.201700104>

6. Y. Xu, C. Zhang, M. Zhou, Q. Fu, C. Zhao, M. Wu, Y. Lei. Highly nitrogen doped carbon nanofibers with superior rate capability and cyclability for potassium ion batteries. Nature Communications. **9**(1), 1720 (2018). <https://doi.org/10.1038/s41467-018-04190-z>

7. J. Ruan, Y. Zhao, S. Luo, T. Yuan, J. Yang, D. Sun, S. Zheng. Fast and stable potassium-ion storage achieved by in situ molecular self-assembling n/o dual-doped carbon network. Energy Storage Materials. **23**, 46-54 (2019). [https://doi.org/10.1016/j.ensm.2019.05.037](https://doi.org/https://doi.org/10.1016/j.ensm.2019.05.037)

8. X. Zhou, L. Chen, W. Zhang, J. Wang, Z. Liu, S. Zeng, R. Xu, Y. Wu, S. Ye, Y. Feng, X. Cheng, Z. Peng, X. Li, Y. Yu. Three-dimensional ordered macroporous metal–organic framework single crystal-derived nitrogen-doped hierarchical porous carbon for high-performance potassium-ion batteries. Nano Lett. **19**(8), 4965-4973 (2019). <https://doi.org/10.1021/acs.nanolett.9b01127>

9. Z. Liu, Y. Zeng, Q. Tang, A. Hu, K. Xiao, S. Zhang, W. Deng, B. Fan, Y. Zhu, X. Chen. Potassium vapor assisted preparation of highly graphitized hierarchical porous carbon for high rate performance supercapacitors. J Power Sources. **361**, 70-79 (2017). [https://doi.org/10.1016/j.jpowsour.2017.06.058](https://doi.org/https://doi.org/10.1016/j.jpowsour.2017.06.058)

10. M. Chen, W. Wang, X. Liang, S. Gong, J. Liu, Q. Wang, S. Guo, H. Yang. Sulfur/oxygen codoped porous hard carbon microspheres for high-performance potassium-ion batteries. Advanced Energy Materials. **8**(19), 1800171 (2018). <https://doi.org/10.1002/aenm.201800171>

11. G. Ma, K. Huang, J.-S. Ma, Z. Ju, Z. Xing, Q.-c. Zhuang. Phosphorus and oxygen dual-doped graphene as superior anode material for room-temperature potassium-ion batteries. Journal of Materials Chemistry A. **5**(17), 7854-7861 (2017). <https://doi.org/10.1039/C7TA01108C>

12. Z. Ju, S. Zhang, Z. Xing, Q. Zhuang, Y. Qiang, Y. Qian. Direct synthesis of few-layer f-doped graphene foam and its lithium/potassium storage properties. ACS Appl Mater Interfaces. **8**(32), 20682-20690 (2016). <https://doi.org/10.1021/acsami.6b04763>

13. H. He, D. Huang, Y. Tang, Q. Wang, X. Ji, H. Wang, Z. Guo. Tuning nitrogen species in three-dimensional porous carbon via phosphorus doping for ultra-fast potassium storage. Nano Energy. **57**, 728-736 (2019). <https://doi.org/https://doi.org/10.1016/j.nanoen.2019.01.009>

14. X. Zhao, Y. Tang, C. Ni, J. Wang, A. Star, Y. Xu. Free-standing nitrogen-doped cup-stacked carbon nanotube mats for potassium-ion battery anodes. ACS Applied Energy Materials. **1**(4), 1703-1707 (2018). <https://doi.org/10.1021/acsaem.8b00182>

15. J. P. Perdew, K. Burke, M. Ernzerhof. Generalized gradient approximation made simple. Phys Rev Lett. **77**(18), 3865-3868 (1996). <https://doi.org/10.1103/PhysRevLett.77.3865>

16. B. Delley. From molecules to solids with the dmol3 approach. The Journal of Chemical Physics. **113**(18), 7756-7764 (2000). <https://doi.org/10.1063/1.1316015>

17. Y. Tian, Y.-j. Liu, J.-x. Zhao, Y.-h. Ding. High stability and superior catalytic reactivity of nitrogen-doped graphene supporting pt nanoparticles as a catalyst for the oxygen reduction reaction: A density functional theory study. RSC Advances. **5**(43), 34070-34077 (2015). <https://doi.org/10.1039/C5RA02585K>
